# Editorial: Translation and implementation of pharmacogenomic testing in daily clinical practice: Considering current challenges and future needs

**DOI:** 10.3389/fphar.2022.1053027

**Published:** 2022-11-11

**Authors:** Wojciech Miltyk, George P. Patrinos, Celine Verstuyft, Marieke Coenen, Alireza Tafazoli

**Affiliations:** ^1^ Department of Analysis and Bioanalysis of Medicines, Medical University of Białystok, Bialystok, Poland; ^2^ Laboratory of Pharmacogenomics and Individualized Therapy, Department of Pharmacy, School of Health Sciences, University of Patras, Patras, Greece; ^3^ Department of Genetics and Genomics, College of Medicine and Health Sciences, United Arab Emirates University, Al-Ain, United Arab Emirates; ^4^ Zayed Center for Health Sciences, United Arab Emirates University, Al-Ain, United Arab Emirates; ^5^ Faculté de Médecine Paris Saclay GH Paris Saclay APHP, Bicetre Hospital, Paris, France; ^6^ Department of Human Genetics, Radboud University Nijmegen Medical Centre, Nijmegen, Netherlands

**Keywords:** pharmacogenomic testing, daily clinical practice, public healthcare systems, pharmacogenetics, pharmacogenomics, PGx, personalised medicine

Although the advancements in pharmacogenomics (PGx) may bring true advantages of personalized medicine into daily clinical practice, the integration and inclusion of the field in routine clinical decision support systems are still very low (Abou Diwan et al., 2019). This is mainly because of intrinsic challenges to the functional prediction of genomic variants in drug-related genes. Responsible genes for drug-metabolizing, transporting, receptors, and targeting were not conserved during the evolution as they encountered various types of xenobiotics and underwent different mutations to be adapted for dealing with such external components in the human body. Also, the existence of structural complexities within pharmacogenes caused the related haplotypes to be hard to catch. Hence, available tools for functional characterization of changes in these genes could not be successful in strongly displaying the consequences of such alteration on drug pharmacokinetics (PK) and pharmacodynamics (PD) (Chang et al., 2021).

Above mentioned reasons not only reduce the speed of PGx investigations and guideline development but also produce several external barriers to the integration of clinical PGx tests into a routine clinical setting. Factors like lower background and expertise for clinical interpretation of PGx test result in physicians and clinicians, lack of particular cost and time benefit instruments and facilities for test implementation through clinical centers, absence of sufficient guidelines for every genomic variant in drug-related genes, no existence of appropriate variant calling tools for many pharmacogenes, no willing and hesitance of insurance parties to cover the tests in clinics, etc. are seen and introduced as the major issues for prevention of combination of PGx and primary care everywhere (Frick et al., 2016).

However, recent years were witness huge motivations and efforts on overcoming such challenges. Several research groups explored the possibility of adding PGx tests as part of clinical decision systems in hospitals and/or private clinical centers (Adesta et al., 2021). Current policies and activities toward the implementation of PGx for various types of patients in different populations investigated and the pros and cons of the tests have been listed as well (Caraballo et al., 2020; Blagec et al., 2022). This special issue aimed to provide an overview of such programs and display the result of related studies on major barriers plus the advancements in the field to reach the goals.

The authors of the Neuropsychiatric and Montelukast article tried to clarify the relation between montelukast and neuropsychiatric in raising adverse events, which resulted in a significant association between neuropsychiatric adverse reactions and montelukast. Such data from real-world samples may add invaluable knowledge to the physicians’ background on PGx and encourage the utilization of results in daily clinical settings (Umetsu et al.). The next article in our special issue investigated the possible effects of *CYP2D6* special genotype and Metoprolol tolerance in Chinese elderly with cardiovascular disorders. The study proved the association between intermediate metabolizers showing lower tolerance and may develop higher incidence of Metoprolol adverse reactions in such patients (Chen et al.). The third paper, thiopurine therapy via *TPMT* and *NUDT15* testing, confirmed the benefits of the implementation of single gene PGx testing, which can guide the transition to a pre-emptive multi-gene testing approach that provides the opportunity to improve clinical care (Goh et al.). The fourth and fifth articles (*DPYD* pre-clinical testing in Switzerland and mini review on genetic associations with severe adverse drug events) explored the prospect of prevention of adverse drug reactions (ADRs) through the utilization of clinical PGx tests and demonstrated the advantages of pre-emptive genotyping on anticipation of ADRs and acceleration of integration of PGx tests into daily primary care (Begré et al.; Wang et al.).

To introduce PGx tests into daily practice, future efforts may focus on offering population-specific pharmacovariant evidence and panel-based sequencing approaches. Widespread access to genomic databases alongside the PGx maps for individuals in a portable format might be also worth consideration.
